# A mutation in the promoter region of *zipA*, a component of the divisome, suppresses the shape defect of RodZ-deficient cells

**DOI:** 10.1002/mbo3.116

**Published:** 2013-08-06

**Authors:** Daisuke Shiomi, Hironori Niki

**Affiliations:** 1 Microbial Genetics Laboratory, Genetic Strains Research Center, National Institute of GeneticsSokendai, 1111 Yata, Mishima, Shizuoka, 411-8540, Japan; 2 Department of Genetics, The Graduate University for Advanced StudiesSokendai, 1111 Yata, Mishima, Shizuoka, 411-8540, Japan

**Keywords:** Cell wall, *Escherichia coli*, microbial cell biology, molecular genetics

## Abstract

RodZ is important for maintaining the rod shape of *Escherichia coli*. Loss of RodZ causes conversion of the rod shape to a round shape and a growth rate slower than that of wild-type cells. Suppressor mutations that simultaneously restore both the growth rates and the rod shape were isolated. Most of the suppressor mutations are found in *mreB*, *mrdA*, or *mrdB*. One of the mutations was in the promoter region of *zipA*, which encodes a crucial component of the cell division machinery. In this study, we investigated the mechanism of the suppression by this mutation. ZipA was slightly but significantly increased in the suppressor cells and led to a delay in cell division. While round-shaped *mreB* and *mrdA* mutants lose cell bipolarity, we found that round-shaped *rodZ* mutants retained cell bipolarity. Therefore, we concluded that a delay in the completion of septation provides extra time to elongate the cell laterally so that the *zipA* suppressor mutant is able to recover its ovoid or rod shape. The suppression by *zipA* demonstrates that the regulation of timing of septation potentially contributes to the conversion of morphology in bacterial cells.

## Introduction

Bacterial cells with a rod shape are composed of two geometrical parts: a central cylinder and hemispherical caps at both ends of the cylinder. Formation of the rod shape is controlled by two distinct modes of synthesis of peptidoglycan: elongation and septation. For elongation, peptidoglycan, which is newly synthesized by more than 10 enzymes (den Blaauwen et al. 2008; Sauvage et al. 2008), is inserted homogeneously into the central cylinder, while peptidoglycan of the cell poles is inert (de Pedro et al. 1997). In *Escherichia coli*, penicillin-binding protein (PBP) 2, encoded by *mrdA*, is a specific enzyme required for the synthesis of the cylindrical wall (Spratt 1975; Spratt and Pardee 1975). It has been proposed that the bacterial actin MreB along with MreC and MreD form a complex with PBP2 and its functional partner RodA, and that the complex plays a role in the synthesis of the cylindrical wall (Kruse and Gerdes 2005; van den Ent et al. 2006). In contrast, septation relies on the bacterial tubulin FtsZ and associated proteins consisting of cell division machinery called the divisome. One of them is PBP3, encoded by *ftsI*, a specific enzyme required for the synthesis of septal peptidoglycan (Spratt 1975; Spratt and Pardee 1975; Begg et al. 1986). It has been thought that these two modes of synthesis of the cell wall are independent of each other. However, it has been shown recently that FtsZ contributes to cell elongation at the midcell and sidewall near the cell poles (Varma et al. 2007; Varma and Young 2009), and that ZipA, a component of the divisome, is required for cell elongation at the midcell which has been called PBP3-independent peptidoglycan synthesis (PIPS) (Nanninga 1991; Potluri et al. 2012). Furthermore, it has been shown that MreB, MreC, MreD, PBP2, and RodA localize to the midcell during the cell division cycle in *E. coli* (Vats and Rothfield 2007; Vats et al. 2009; van der Ploeg et al. 2013), suggesting that these components of cell elongation can play some role at the division site. Thus, the separation of the synthesis of peptidoglycan for elongation and division is not as clear as previously thought. In any case, as in eukaryotes, cytoskeletal proteins in prokaryotes are involved in both elongation and septation, and hence the determination of morphology is possibly through the regulation of localization and the activity of enzymes such as PBP2 and PBP3 that synthesize peptidoglycan (Dye et al. 2005; Kruse et al. 2005; van den Ent et al. 2006; Vats et al. 2009).

RodZ is also an important determinant for cell shape (Shiomi et al. 2008; Alyahya et al. 2009; Bendezu et al. 2009). *Escherichia coli* cells lacking either functional MreB or RodZ become round, although the *mreB* mutant cell is larger than the *rodZ* mutant (Shiomi et al. 2008). The bacterial actin MreB is involved in the determination of cell bipolarity as well as in cell elongation (Nilsen et al. 2005; Shih et al. 2005; Pradel et al. 2007). Defects of *mreB* in *E. coli* cause the mutant cells to become round without “poles” instead of rod shaped with bipoles (Shih et al. 2005). Although RodZ colocalizes with MreB in vivo (Shiomi et al. 2008; Alyahya et al. 2009; Bendezu et al. 2009), and they interact with each other in vitro (van den Ent et al. 2010), some of their functions are different (Shiomi et al. 2008).

To investigate the function of RodZ, suppressors were isolated that grew faster and were found to also restore the rod shape (Shiomi et al. 2013). Whole genomic sequencing of these mutants identified suppressor mutations. Twenty-six of the 29 suppressor mutations were in *mreB*, *mrdA*, or *mrdB*, whose gene products are involved in peptidoglycan synthesis for cell elongation (Shiomi et al. 2013). These mutations change the properties of the MreB protein so that the suppressors can synthesize the cylindrical peptidoglycan without RodZ. Unexpectedly, another mutation was found in the upstream region of the *zipA* gene, encoding an essential membrane protein involved in cell division to stabilize the assembled Z ring (Hale and de Boer 1997; Pichoff and Lutkenhaus 2002). Here, we address the mechanism of suppression by *zipA* to restore the cell shape of the *rodZ* deletion mutant.

## Results

### Recovery of cell shape by a suppressor mutation of *zipA*

**Figure 1:**
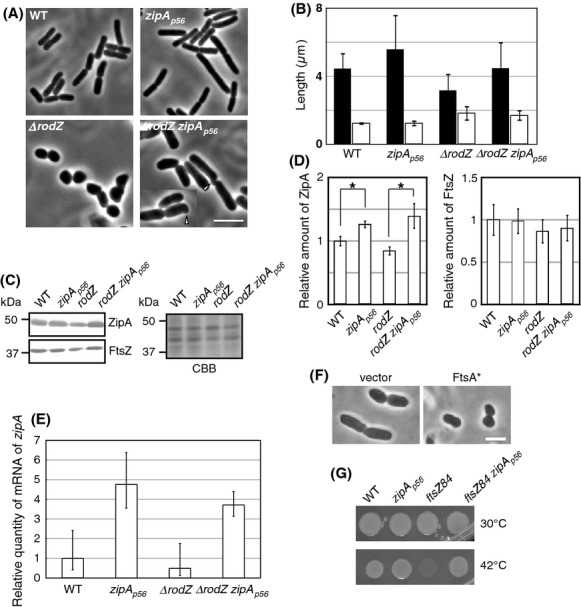
Suppression of the *rodZ* mutant by the *zipA_p56_* mutation. (A) Phase contrast images of DS645 (WT), DS554 (*zipA_p56_*), DS679 (Δ*rodZ*), and DS631 (Δ*rodZ*, *zipA_p56_*), which were grown in L broth at 37°C. Scale bar represents 2.5 μm. (B) Mean cell lengths and SD of the length (black) and short (white) axes of DS645 (*N* = 108), DS554 (*N* = 133), DS679 (*N* = 281), and DS631 (*N* = 331). (C) Immunoblotting analysis of ZipA or FtsZ protein using an antibody against ZipA (upper) or FtsZ (bottom). A CBB-stained gel (right) confirmed that the same amount of the sample among strains was applied to SDS-PAGE. (D) Each band was normalized with the intensity of the band derived from ZipA or FtsZ in WT. The amounts of ZipA in WT or in *rodZ* mutant cells carrying the *zipAp56* mutation was significantly higher than those in WT or in the *rodZ* mutant. The asterisk shows *P* value < 0.05 as determined by a *T*-test. (E) Relative quantity of mRNA of *zipA* in DS645 (WT), DS554 (*zipA_p56_*), DS679 (Δ*rodZ*), and DS631 (Δ*rodZ*, *zipA_p56_*) as calculated by real-time PCR shown in (Fig. S4). The quantity was normalized by the amount of mRNA of *zipA* in WT. (F) Phase contrast images of DS631 (Δ*rodZ*, *zipA_p56_*) carrying pWM2784 (vector) or pWM2787 (ftsA*). Scale bar represents 2 μm. (G) Suppression of thermosensitivity of *ftsZ84* by introduction of *zipA_p56_*. Five microliter of overnight culture of DS708 (WT), DS709 (*zipA_p56_*), DS710 (*ftsZ84*), and DS711 (*fts84*, *zipA_p56_*) were spotted onto L plates and incubated at 30 or 42°C.

We refer to the suppressor mutation in the upstream region of *zipA* as *zipA*_*p56*_ hereafter. After whole-genome sequencing of the suppressor, we confirmed the mutation by resequencing of the chromosomal region. Furthermore, we transferred the *zipA*_*p56*_ mutation to the wild-type and to the *rodZ* deletion mutant using transduction of P1 vir phage. The suppressor strain DS631 (Δ*rodZ zipA*_*p56*_) was isolated as one of the spontaneous mutants that grew faster than the *rodZ* deletion mutant in rich medium (Table S1). This suppressor mutation simultaneously suppressed the defect in the cell shape as seen with other suppressor mutations (Fig. 1A) (Shiomi et al. 2013). We analyzed the cell size of DS679 (Δ*rodZ*) and DS631 (Δ*rodZ*, *zipA*_*p56*_) (Fig. 1B). The cell width did not significantly change between DS679 and DS631: 1.83 ± 0.45 and 1.70 ± 0.32 μm, respectively, which was wider than that of the wild type (1.24 ± 0.09 μm). However, the cell length of DS631 (4.47 ± 1.70 μm) was increased compared with DS679 (3.17 ± 0.98 μm), and was similar in length to the wild type (4.45 ± 0.95 μm). The suppressor mutation *zipA*_*p56*_ restored not only cell growth but also the cell shape of the *rodZ* mutant.

Previously, we showed that most of the *mreB*, *mrdA*, and *mrdB* mutants isolated as suppressors of *rodZ* in rich medium could not recover the cell growth and shape of the *rodZ* mutant in minimal medium (Shiomi et al. 2013). Likewise, the suppressor strain carrying *zipA*_*p56*_ was not rod shaped but round in minimal medium, while the wild-type strain remained rod shaped in the same medium (Fig. S1), indicating that the effect of the suppressor mutations is dependent on the medium. We also have shown that suppressor strains carrying a suppressor mutation in *mreB*, *mrdA*, or *mrdB* grow at low temperatures and show swarming ability while the *rodZ* mutant doesn't (Shiomi et al. 2013). The *zipA*_*p56*_ mutation restored not only growth and cell shape at low temperatures but also the swarming ability of the *rodZ* mutant (Figs. S2A, S2B, and S3).

### Increased expression of ZipA by the *zipA*_*p56*_ mutation

The mutation *zipA*_*p56*_, which is a base substitution from T (thymine) to A (adenine), is located 56 bases upstream of the initiation codon of *zipA*. The position is not in the putative −10 and −35 promoter regions, which are located at 185 and 211 bases upstream of the *zipA* gene, respectively (Hale and de Boer 1997). Nevertheless, we thought that the mutation would be in a regulatory region and affect the amount of cellular ZipA. Indeed, immunoblotting analysis revealed that the amount of ZipA was slightly but statistically increased in both DS554 (*zipA*_*p56*_) and DS631 mutants (Δ*rodZ zipA*_*p56*_) compared with DS645 (wild-type) and DS679 (Δ*rodZ*): a 1.26-fold and 1.64-fold increase, respectively (Fig. 1C and D), while the amount of FtsZ was comparable in these strains (Fig. 1C and D). Measurements of transcribed mRNA by real-time PCR revealed that the amount of mRNA of *zipA* was statistically higher in DS554 (*zipA*_*p56*_) and DS631 (Δ*rodZ zipA*_*p56*_) cells than in DS645 (wild-type) or DS679 (Δ*rodZ*) (Fig. 1E and Fig. S4). In addition, *rodZ* mutant cells harboring a multicopy plasmid encoding *zipA* expressed from arabinose promoter P_BAD_ (pDS1019) grew faster than mutant cells carrying an empty vector (Fig. S5A), and were restored the cell shape (Fig. S5B). The amount of *zipA* may be increased by the gene dosage effect. These results indicate that the suppressor mutation was within the regulatory region of the *zipA* gene and caused increased expression of *zipA*.

Overproduction of ZipA, even a twofold, is toxic to cells, resulting in filaments in wild-type cells (Fig. S5B) (Geissler et al. 2003). A mutant FtsA (FtsA-R286W), known as FtsA*, abolishes the negative effect of excess ZipA by an unknown mechanism, and long-term lethal filamentation is inhibited (Geissler et al. 2003). By analogy, FtsA* should abolish the effect of *zipA*_*p56*_ on the suppression of the *rodZ* mutant. A vector plasmid or a plasmid encoding ftsA* was introduced into the suppressor strain DS631 (Δ*rodZ*, *zipA*_*p56*_). Cells carrying a plasmid encoding ftsA* became oval or round (Fig. 1F), which was very similar in shape to the *rodZ* cells, suggesting that the “negative” effect of increased ZipA was canceled by FtsA*. In addition, the growth rate of DS631 carrying ftsA* was slower than that of DS631 carrying an empty vector (44 vs. 36 min). Thus, upregulation of *zipA* was sufficient to suppress the deficiency in cell shape maintenance.

The *zipA*_*p56*_ mutation is also located in a putative regulatory region of the *cysZ* gene, whose function is unknown. So far, there is no evidence of relationship between CysZ and FtsA. Growth of *rodZ* cells expressing extra *cysZ* from the arabinose promoter P_BAD_ was similar to that of the *rodZ* cells harboring pBAD24 (data not shown). In addition, as we showed above, the gene dosage effect of ZipA caused suppression of the *rodZ* cells and production of FtsA* in the DS631 antagonizes the effect of the suppression. Thus, we concluded that the suppression by the *zipA*_*p56*_ mutation occurs through the regulation of expression of *zipA*, and not *cysZ*.

### *zipA*_*p56*_ can suppress temperature-sensitive growth of cells carrying the *ftsZ84* mutation

It has been reported that higher expression of *zipA* can suppress temperature sensitivity of the *ftsZ84* mutation by stabilizing the labile FtsZ84 ring structure (RayChaudhuri 1999). To confirm whether a slight upregulation of *zipA* has the same effect, the *zipA*_*p56*_ mutation was introduced into wild-type (WM1074) or *ftsZ84* mutant (WM1125) cells, yielding DS709 (*zipA*_*p56*_) and DS711 (*ftsZ84*, *zipA*_*p56*_). As seen in Fig. 1(G), DS711 cells (*ftsZ84*, *zipA*_*p56*_) grew on L plates at the restrictive temperature of 42°C, while WM1125 cells (*ftsZ84*) failed to form colonies. These results indicate that the increased amount of ZipA expressed in strains carrying the *zipA*_*p56*_ mutation is sufficient to stabilize the assembly of FtsZ84.

### The *zipA*_*p56*_ mutation could not restore viability to the *mreB* or *mrdA* deletion mutants

It has been shown that overproduction of FtsZ can restore viability to round-shaped *mreB* or *mrdA* mutants (Bendezu and de Boer 2008). Thus, we examined whether the *zipA*_*p56*_ mutation rescued the lethality and defect in the cell shape of the *mreB* or *mrdA* mutants. The *zipA*_*p56*_ mutation did not suppress the lethality of either of the strains in L broth (Fig. S6A). We could not conclude whether the *zipA*_*p56*_ mutation restored their shape in L broth. The *mreB* or *mrdA* mutants are viable in minimal synthetic media. In M9 minimal medium, the *zipA*_*p56*_ mutation did not suppress the cell shape defect of *mreB* or *mrdA* mutants or that of the *rodZ* mutant (Fig. S6B). Thus, differences in the causes of conversion from rod cells to round cells between the *mreB* or *mrdA* mutants and the *rodZ* mutant were evident.

### Elongation of *rodZ* mutant cells during the cell division cycle

**Figure 2:**
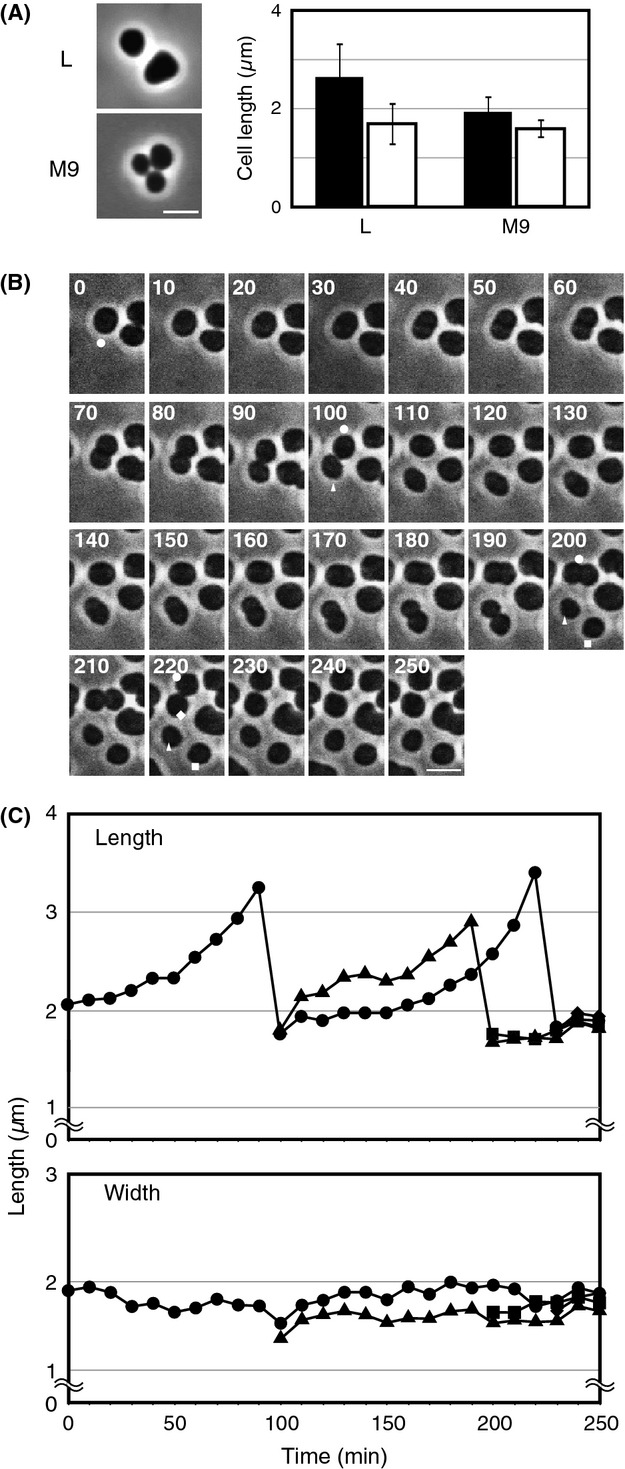
Cell division of Δ*rodZ* cells. (A) Morphology of DS290 (Δ*rodZ*) cells grown in L broth (*N* = 329) or M9 medium supplemented with glucose (*N* = 276) (left). The means and standard deviations (SD) of length shown in black, and of width shown in white of cells grown in M9 medium are at right. Scale bar represents 2.5 μm. (B) Time-lapse images of cell division of Δ*rodZ* cells. Cells were grown to mid log phase in M9 medium supplemented with 0.25% glucose and then mounted in agar. Images were taken at 2 min intervals, and numbers shown at the top left in each image represent time (min). A cell marked by a circle started elongation at time 0 and divided into two cells marked by a circle and triangle at time 100. A cell marked by a circle divided into two cells marked by circle and diamond at time 220 min, while a cell marked by a triangle divided into two cells marked by triangle and square at time 200 min. Scale bar represents 2.5 μm. (C) Measurement of lengths of long (top) and short (bottom) axes. Each symbol represents the same marked cells in (B).

It seemed that suppression of the *rodZ* mutant by overproduction of ZipA was related to cell division. We thus examined the manner of division of the *rodZ* mutant. Cells were cultured in M9 synthetic medium, because cells in this medium were more round than those in rich medium, although oval-shaped cells were observed in both synthetic and rich media (Fig. 2A). We carried out time-lapse observations of *rodZ* mutant cells grown in synthetic medium every 2 min (Fig. 2B). The cells did not swell, but they elongated along the long axis of the cell and divided at the midcell (Fig. 2B). Daughter cells were almost completely round and then elongated gradually. As a result, round daughter cells became oval. To confirm the lateral elongation of cells, we measured the length and width of dividing cells. As seen in Fig. 2(C), the width was almost constant (mean: 1.7 μm), while the length continuously elongated and reached twofold of the width (maximum length: 3.4 μm) (Fig. 2C). These results indicate that cells elongated unidirectionally while the cell width was constant. In addition, this result suggests that, even though the *rodZ* mutant cells are round, the cells retain cell bipolarity; that is, a determinant of the direction of elongation.

### Directional oscillation of Min protein in round cells

**Figure 3:**
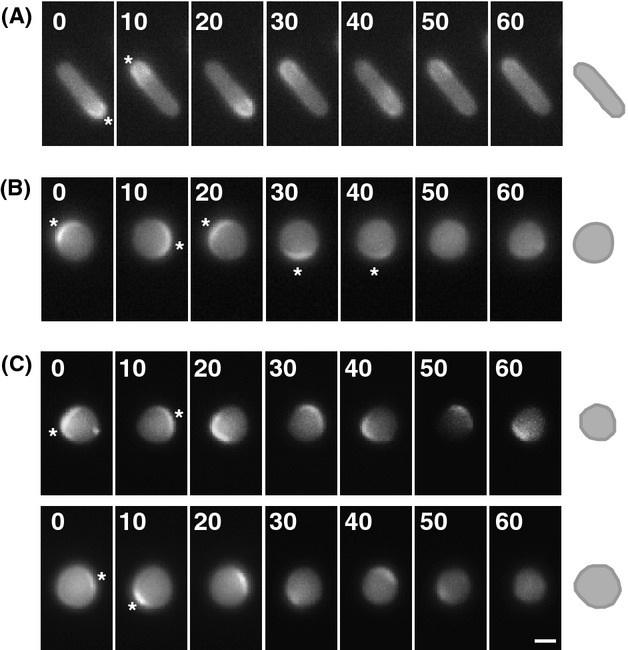
Oscillation of MinD fused with GFP. BW25113 (A), BW25113 treated with A22 (B), and DS290 (C). Cells producing GFP-MinD were grown in M9 glucose in the presence of 10 μmol/L IPTG. Cells were mounted in M9 glucose containing 2% agarose. Images were taken at 10 sec intervals. Cells with corresponding schematic depictions are shown. Asterisks represent localization of GFP-MinD. Scale bar represents 1 μm.

To confirm that *rodZ* mutant cells retain cell bipolarity, we observed the oscillation of the MinD protein. Min proteins, which consist of MinC, MinD, and MinE, oscillate between the end poles to control assembly of FtsZ at the midcell, and then Z ring formation is inhibited at the cell poles (Raskin and de Boer 1999; Hale et al. 2001). Indeed, GFP-MinD rapidly oscillated from one pole to the other in a rod-shaped wild-type *E. coli* cell (Fig. 3A). It has been previously shown that oscillations of MinD are often random in spherical *mrdB* or *mreB* mutant cells (Begg and Donachie 1998; Corbin et al. 2002; Shih et al. 2005). Similar results were obtained in spherical wild-type cells after the addition of A22 that inhibits binding of ATP to MreB (Fig. 3B). Random oscillation patterns suggest a loss of polarity in *mrdB*, *mreB* round cells, or spherical wild-type cells by A22. However, oscillation of MinD was not random in spherical *rodZ* mutant cells in which a polar cap of GFP-MinD was observed (Fig. 3C). The visible polar cap rapidly moved to the opposite side, and the direction of oscillation of GFP-MinD was constant in the *rodZ* cells. This movement of GFP-MinD supports the hypothesis that the *rodZ* cells retain a fixed polarity and can divide symmetrically at the midcell.

### Cell elongation by arrest of cell division

**Figure 4:**
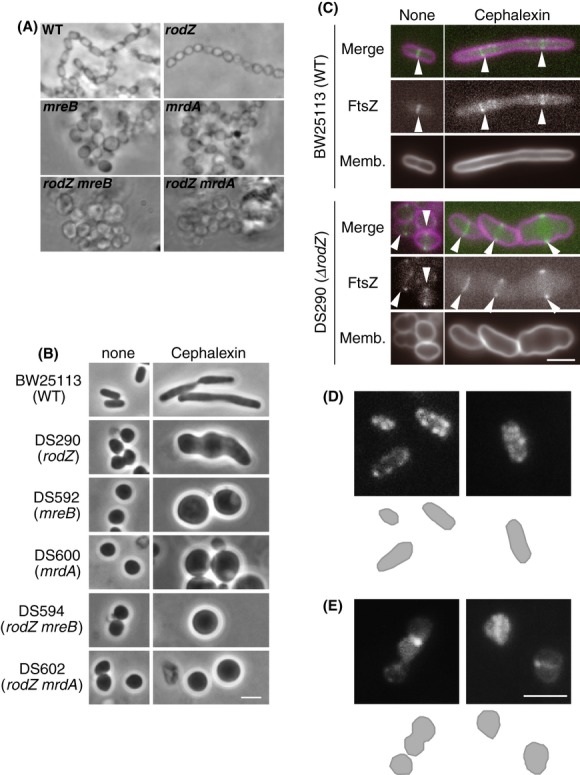
Morphology of cells treated with cephalexin. (A) Cell division of BW25113 (WT), DS290 (Δ*rodZ*), DS592 (Δ*mreB*), DS600 (Δ*mrdA*), DS594 (Δ*rodZ*, Δ*mreB*), or DS602 (Δ*rodZ*, Δ*mrdA*). Cells were grown in M9 medium supplemented with 0.25% glucose overnight and then mounted in M9 glucose containing 30% gelatin and incubated at 30°C overnight. (B) Phase contrast images of BW25113, DS290, DS592, DS600, DS594, or DS602 cells grown in M9 glucose in the absence or presence of 10 μg/mL cephalexin for 1.5 h. Scale bars represent 2.5 μm. (C) BW25113 or DS290 cells producing FtsZ-GFP were grown in M9 glucose in the absence or presence of 10 μg/mL cephalexin for 1.5 h. Membrane was stained by FM4-64. Arrowheads represent the Z ring. (D, E) Visualization of nascent peptidoglycan (d-Ala-d-Ala). Sacculi of BW25113 (D) and DS290 (E) were prepared and incubated with 10 μg/ml vancomycin labeled with BODIPY-FL for 1 h in the dark. Fluorescent pictures with corresponding schematic depictions are shown. Scale bar is 2 μm.

If the direction of cell elongation is transmitted to daughter cells, division planes that are perpendicular to the axis should be parallel to each other in daughter cells, and then descendants would form chains. In order to observe cells, we used a gelatin-mounting method (Mason and Powelson 1956; Yamaichi and Niki 2004) to avoid completely separating cells from each other after cell division. In fact, newly divided cells were arranged linearly when *rodZ* mutant and wild-type cells were mounted in medium containing gelatin (Fig. 4A). In contrast, a cluster was formed by round cells of the *mreB* mutant, which lose polarity after cell division (Shih et al. 2005) (Fig. 4A).

Furthermore, the effect of inhibition of cell division on cell morphology was examined to confirm the fixation of the direction of cell elongation. Cephalexin, which inactivates PBP3 that synthesizes peptidoglycan at the septum during cell division, inhibits cytokinesis of bacteria and results in elongation of the cells (Pogliano et al. 1997; Weiss et al. 1997) (Fig. 4B). On the other hand, when the division of the *rodZ* mutant cells was inhibited by cephalexin, the mutant cells became warped filaments with swollen balloons, extending along the long axis (Fig. 4B). It is currently unclear what constitutes these balloons. In addition, cells treated with cephalexin were wider than untreated cells. Interestingly, round-shaped *mreB* mutant cells became larger spheres after the addition of cephalexin (Fig. 4B). It seems likely that the *mreB* mutant cells swelled as a whole because they had lost polarity for the direction of cell elongation and expanded in all directions. Likewise, cells with both *rodZ* and *mreB* deleted formed clusters after cell division and became larger spheres upon inhibition of cell division (Fig. 4A and B).

FtsZ localizes to the division site and forms a ring-like structure called the Z ring (Bi and Lutkenhaus 1991; Ma et al. 1996). GFP-tagged FtsZ (FtsZ-GFP) formed a Z ring at the division plane located at the midcell of short cells or at the quarter sites of elongated cells in the wild-type (Fig. 4C). FtsZ-GFP was still localized at the midcell of the *rodZ* mutant cells in the absence and presence of cephalexin, and additionally, FtsZ rings were perpendicular to the long axis as in the wild type (Fig. 4C). Thus, directional elongation by inhibition of cell division indicates that constant directionality of the long axis clearly exists in the *rodZ* mutant cells. These results also indicate that inhibition of cell division of the *rodZ* mutant results in recovery of the cell shape to a rod.

### Deletion of PBP2 in the *rodZ* mutant

It has been thought that lateral peptidoglycan synthesis requires PBP2 (Spratt 1975; Spratt and Pardee 1975), and that PBP2 is usually indispensable for cell growth in *E. coli*. However, PBP2 should be dispensable in the *rodZ* mutant because, presumably, lateral elongation of the cell wall is unnecessary if the *rodZ* mutant consists of only polar caps. A deletion mutant for the *mrdA* gene encoding PBP2 can grow on synthetic medium, but not on nutritionally rich medium (Bendezu and de Boer 2008) (Fig. S6A). Cells deleted for both *rodZ* and *mrdA*, as well as cells deleted for only *mrdA*, failed to grow on nutritionally rich medium, suggesting that the *rodZ* mutation could not suppress the growth deficiency caused by the *mrdA* mutation (Fig. S6A). The inability to delete *mrdA* from the *rodZ* mutant may be because: (1) the *rodZ* mutant retains a very small portion of cylindrical peptidoglycan that is synthesized by PBP2, and/or (2) PBP2 plays a role in other essential functions such as the establishment of cell polarity. As seen in Figure 4(A and B), cells deleted for *mrdA* or both *mrdA* and *rodZ* formed aggregates after division and became larger spherical cells, suggesting that cells with deleted *mrdA* lost cell polarity.

### Active peptidoglycan synthesis only at the midcell of the *rodZ* mutant

The elongation of a rod cell is dependent on the synthesis of lateral peptidoglycan at the cylindrical part. We predicted that the active site of peptidoglycan synthesis for cell growth should be changed in the round cells of the *rodZ* mutant. Fluorescent vancomycin was used to detect the active sites of peptidoglycan synthesis (Varma et al. 2007; Varma and Young 2009). Vancomycin specifically binds to terminal d-alanyl-d-alanine moieties of peptides that are to be closely linked to sugar chains in alternating *N*-acetylglucosamine and *N*-acetylmuramic acid. Even though d-Ala-d-Ala are only present in *E. coli* cells as 1% or less of total muropeptides (Glauner et al. 1988), Young and colleagues applied a vancomycin-labeling method of isolated sacculi to visualize the sites of peptidoglycan insertion (Varma et al. 2007; Varma and Young 2009). Isolated single giant molecules of the peptidoglycan layer, which maintains the rod shape of living cells, were labeled by fluorescent vancomycin. Fluorescent spots were distributed as spirals along the lateral side of rod-shaped sacculi (Fig. 4D). However, fluorescence in the *rodZ* mutant was localized only at the midcell plane and septum in dividing cells (Fig. 4E), indicating that peptidoglycan synthesis actively occurs at the midcell of the *rodZ* mutant. This result is consistent with the assumption that the lateral part of the rod cell is remarkably shortened or lost in the *rodZ* mutant.

### Delay of cytokinesis by increased ZipA

**Figure 5:**
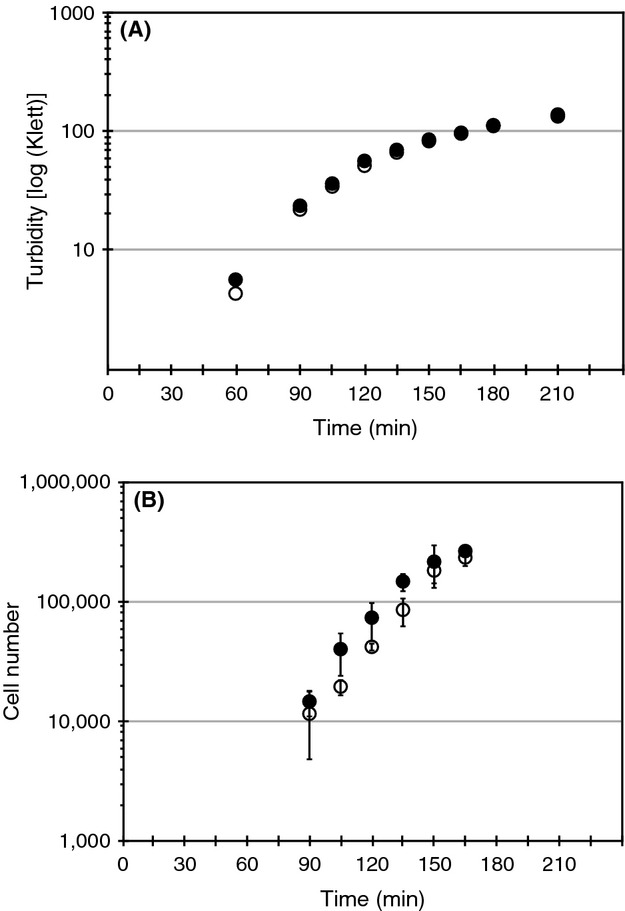
Analyses of the suppressor mutant (*zipA_p56_*). (A, B) Turbidity (A) and cell numbers (B) of DS645 (WT) (filled circles) and DS554 (*zipA_p56_*) (circles) grown in L broth at 37°C. Turbidity was measured as the Klett value, and cells were harvested to measure cell number at the indicated times.

So far, we found that inhibition of cell division of the *rodZ* mutant resulted in its apparent rod shape (Fig. 4C). It has been previously shown that overproduction of ZipA results in elongation of a cell, possibly because division is inhibited (Geissler et al. 2003). These results allow us to hypothesize that suppression of the *rodZ* mutant by *zipA*_*p56*_ is due to inhibition or delay of cell division of the mutant. As shown in Figure 2(B), the *rodZ* cells elongate laterally during the division process. Thus, we examined whether cell division of *rodZ*^+^ cells is inhibited by *zipA*_*p56*_. Cell growth was measured by the following two independent methods: measurement of the turbidity of the culture medium that reflects an increase in cell mass, and cell counting using a flow cytometer. The profiles of turbidity were similar between wild-type and *zipA*_*p56*_ (Fig. 5A), and the mass doubling times were the same (Table S1). The similar increase in cell mass between wild-type and *zipA*_*p56*_ indicates that both strains elongated at almost the same rate. On the other hand, differences were observed in the increase in cell number between wild type and *zipA*_*p56*_ (Fig. 5B). The number of wild-type cells increased more rapidly than that of the *zipA*_*p56*_ cells. This inconsistency between cell mass and cell number can be explained by the *zipA*_*p56*_ cells taking a longer time to divide into daughter cells compared to the wild-type, while their cell volume increased at a similar rate. Thus, these results indicate that the *zipA*_*p56*_ mutation leads to a delay of cell division. Consequently, we propose that the *zipAp56* mutation causes a delay of cell division in the *rodZ* mutant, and hence the mutant becomes apparently rod shaped.

## Discussion

### Mechanism of suppression of the *rodZ* mutant by *zipA*_*p56*_

**Figure 6:**
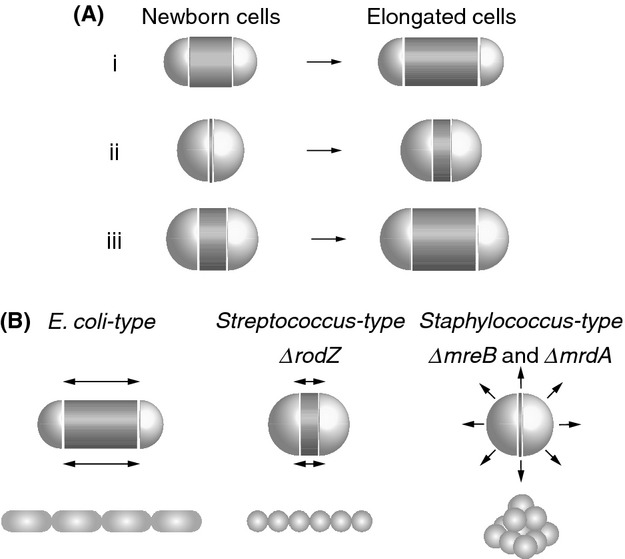
Morphological change of bacterial cells from round to rod. (A) Proposed mechanism of restoration of the rod shape of Δ*rodZ* cells by increased ZipA. (1) Rod-shaped cells of *Escherichia coli* consist of polar caps and a central cylinder, retain cell polarity, and elongate laterally. Peptidoglycan is synthesized at the entire central cylinder so that cells elongate laterally until the cell length is twice as long as a newborn cell. Polar caps are derived from septa, and the peptidoglycan is synthesized during cell division. After that, polar caps inert with respect to synthesis of the peptidoglycan layer (de Pedro et al. 1997). (2) Newly synthesized peptidoglycan is inserted at the midcell in Δ*rodZ* cells so that a round cell gradually elongates along a single axis. Synthesis of the peptidoglycan layer at septum is efficient in forming ovoid-shaped cells. After cell septation, newborn cells are round shaped. (3) Newly synthesized peptidoglycan is inserted at the midcell in Δ*rodZ* cells producing extra ZipA so that cells apparently elongate laterally. The time of cell division is delayed compared with *rodZ* cells so that the cells continue to elongate. Dark gray zones indicate regions where peptidoglycan is actively synthesized. (B) Schematic illustrations of increased cell volume of rod-shaped or round-shaped cells. Rod-shaped *E. coli* cells grow at the central cylinder. Ovoid-shaped *Streptococcus* cells grow at septum while round-shaped *Staphylococcus* cells swell. Black arrows indicate the direction of increase of cell volume. The increase in cell volume of Δ*rodZ* resembles that of *Streptococcus*, while those of Δ*mreB* or Δ*mrdA* resemble that of *Staphylococcus*. Dark gray zones indicate regions where peptidoglycan is actively synthesized. *Escherichia coli* (WT), Δ*rodZ*, and *Staphylococcus* cells retain cell polarity, while *E. coli* Δ*mreB*, Δ*mrdA*, and *Staphylococcus* cells lose polarity as shown at the bottom.

Morphological mutations that convert rod-shaped *E. coli* to round shaped have been found in some genes, including *mreB*, *mrdA*, and *rodZ*. The former two mutations result in relatively larger round cells with defects in the maintenance of cell polarity (Begg and Donachie 1998; Corbin et al. 2002; Shih et al. 2005) (Fig. 4A and B). On the other hand, the *rodZ* round cell may still have a defined single cell polarity and elongates laterally, but probably with a shortened central cylinder of a normal rod cell or squat rod (Shiomi et al. 2009) (Fig. 6A).

An excess amount of ZipA perturbs the physiological function of the Z ring in the divisome and hence inhibits or delays the closure of the divisome for cytokinesis (RayChaudhuri 1999; Geissler et al. 2003). Inhibition of cell division by the *zipA*_*p56*_ mutation could give enough time for “lateral” peptidoglycan synthesis at the midcell to elongate the cell, resulting in recovery of a rod shape (Fig. 6A). Thus, this “negative” effect of ZipA on the Z ring results in elongation of the *rodZ* mutant to recover the rod shape.

How can overproduced ZipA delay cell division? Because ZipA stabilizes the Z ring possibly through direct interaction or indirect effects through ClpXP (RayChaudhuri 1999; Pazos et al. 2013), and thus overproduced ZipA would overstabilize the Z ring, which can cause a delay in cell division.

Synthesis of the lateral cell wall to maintain the rod shape is executed by the elongasome, which is a protein complex that includes PBP2 and MreB (den Blaauwen et al. 2008). In the *rodZ* mutant, the activity of the elongasome may be reduced. In the *rodZ*, *zipA*_*p56*_ mutant, presumably elongasome activity is prolonged at the Z ring during the duration of cell division, and thus cells look rod shaped. This effect of the elongasome can be expected only in the *rodZ* mutant, but not in the *mreB*, *mrdA*, or presumably the *mrdB* mutant. Thus, RodZ is required for the elongasome to work away from the septum but is not required for it to work at the septum.

Moreover, it has recently been reported that FtsZ and ZipA are required for preseptal peptidoglycan synthesis at the septum, known as PIPS in *E. coli* as well as in *Caulobacter crescentus* (Aaron et al. 2007; Varma and Young 2009; Potluri et al. 2012). Peptidoglycan in the *rodZ* mutant is mainly synthesized at the septum (Figs. 4E and 6A). Thus, overproduction of ZipA might activate peptidoglycan synthesis at the septum via PIPS. It has been suggested that some of the components of the elongasome are involved in PIPS (Potluri et al. 2012; van der Ploeg et al. 2013). It seems likely that in the *rodZ*, *zipA*_*p56*_ mutant, both activities of the elongasome and PIPS cooperatively help round cells to recover the rod shape.

Our results suggest an indirect relationship between RodZ and FtsZ. Bendezu et al. (2009) have shown a genetic interaction; that is, overproduced FtsZ improves the growth of the *rodZ* mutant, and *rodZ* cells overproducing FtsZ are not round. Although the mechanisms behind these phenomena are unclear, the results suggest relationships between RodZ and FtsZ-induced cell division.

### Analogy between round-shaped *E. coli* and other round-shaped bacteria

The manner of cell growth of the *rodZ* mutant is analogous to that of the ovococcus bacteria, *Streptococcus pneumoniae*, and *Lactococcus lactis*, as follows: (1) round or ovoid cells, (2) retainment of polarity, and (3) active peptidoglycan synthesis only at the midcell but not within caps (Fig. 6B). Interestingly, inhibition of cell division of ovoid-shaped *Lactococcus lactis* results in rod-shaped cells (Perez-Nunez et al. 2011). This transition from ovoid cells to rod is similar to the mechanism of recovery of the cell shape in the *rodZ* mutant by the *zipA*_*p56*_ mutation. Thus, the *rodZ* mutant can be referred as a “pseudo-ovoid” shaped mutant.

In contrast, the manner of cell growth of the *mreB* or *mrdA* mutants is analogous to staphylococcus-type bacteria. Mutant cells increased as a whole (Figs. 4B and 6B), and larger round cells were divided into smaller daughter cells as seen in *Staphylococcus aureus* (Tzagoloff and Novick 1977). In addition, mutants of *mreB* and *mrdA* formed in grape-like clumps (Fig. 4A). The formation of the grape-like clump after cell septation indicates that the planes of cytokinesis are not constant in each cell. MinD moves often randomly in *mreB* mutants (Begg and Donachie 1998; Corbin et al. 2002; Shih et al. 2005). Thus, the *mreB* and *mrdA* mutants do not retain cell bipolarity correctly. Although both RodZ and MreB are colocalized to maintain the rod shape, each plays a different role in maintaining cell shape.

### Cell shape and cell growth

The surface-to-volume ratio is one of the important parameters that characterizes prokaryotes. Because of their smaller size than eukaryotic cells, bacteria have a large surface-to-volume ratio. This guarantees that nutrients and metabolites can be easily transported between all parts of the cell. When the cell volume is equal between a round cell and a rod cell, the surface-to-volume ratio is naturally larger in the rod cell. This suggests that a rod shape should be advantageous to cell growth for relatively larger bacteria. As shown in a previous study (Shiomi et al. 2013) and in this study, recovery of the defect in growth is always accompanied by conversion of a round cell to a rod cell. Furthermore, whatever the suppressors of the *rodZ* mutation are, recovery of the rod shape restores the cold-sensitive growth and swarming inability of the *rodZ* mutant.

Indeed, the cell volume of streptococci and staphylococci is smaller than that of *E. coli*. The conversion of a rod cell to a round cell in *E. coli* means a reduction in the surface-to-volume ratio. Thus, only one mutation of the *rodZ* gene causes a metabolic revolution in the ability to utilize some carbon sources and other metabolites (Ito et al. 2005) and subsequently results in growth deficiency. Mutating only one base pair, such as in the promoter of the *zipA* gene (*zipA*_*p56*_) and *mreB* gene, would be a relatively simple way to recover the rod shape. Such simple methods might be utilized to convert cell shapes from round to rod.

## Experimental Procedures

### Bacterial strains and growth media

All strains are derivatives of *E. coli* K-12 and are listed in Table S2. BW25113 (Datsenko and Wanner 2000) is the wild-type strain. WM1074 (MG1655 derivative) and WM1125 (WM1074 *ftsZ84*) (Shiomi and Margolin 2007) are gifts from Dr. William Margolin (University of Texas Medical School at Houston). DS290 (BW25113: Δ*rodZ*::*kan*) was constructed by P1 transduction, transferring Δ*rodZ*::*kan* prepared from JW2500 (Δ*rodZ*::*kan*) to BW25113 (Shiomi et al. 2013). Plasmid pCP20 carrying the yeast Flp recombinase (FLP) and ampicillin resistant (Amp^R^) genes were used to transform DS290 (Kan^R^). The resulting transformants were incubated at 42°C and selected as clones that became Amp^S^ and Kan^S^, yielding DS343 (Δ*rodZ*). JD14320 and JD16067 are W3110 derivative strains that are cis diploid mutants carrying a mini-Tn10Kan insertion in the *mreB* and *pbp2* genes, respectively (Miki et al. 2008). DS592 (BW25113: Δ*mreB*::*Tn10kan*), DS594 (BW25113: Δ*rodZ* Δ*mreB*::*Tn10kan*), DS600 (BW25113: Δ*mrdA*::*Tn10kan*), and DS602 (BW25113: Δ*rodZ*, Δ*mrdA*::*Tn10kan*) were constructed by P1 transduction, transferring Δ*mreB*::*Tn10kan* or Δ*mrdA*::*Tn10kan* prepared from JD14320 (Δ*mreB*::*Tn10Kan*) or JD16067 (Δ*mrdA*::*Tn10Kan*) to BW25113 or DS343, respectively. The transductants were selected on M9 plates containing 0.25% glucose at 37°C. Cells were grown in L broth or M9 medium containing 0.25% glucose at indicated temperatures. Kanamycin (15 μg/mL), ampicillin (20 or 100 μg/mL), and chloramphenicol (20 μg/mL) were added to the culture medium or plates when necessary.

### Plasmids

All plasmids used in this study are listed in Table S2. *ftsZ*, *minD*/*minE*, *zipA*, and *cysZ* genes were amplified using BW25113 cells as a template and high fidelity DNA polymerase KOD-plus (TOYOBO, Osaka, Japan). The PCR products were cloned as SacI-XbaI, SacI-HindIII, and NcoI-HindIII fragments into pDSW210, pDSW209, and pBAD24 to yield pDS156 (pDSW210-*ftsZ*-*gfp*), pDS209 (pDSW209-*gfp*-*minD*/*minE*), pDS1019 (pBAD24-*zipA*), pDS1391 (pBAD24-*cysZ*), respectively.

### Isolation of suppressors and genomic sequencing

Details were provided previously (Shiomi et al. 2013). Independent single colonies of DS6 (Δ*rodZ*) were grown in L broth containing kanamycin at 37°C. Cells were transferred every day to fresh L broth containing kanamycin and kept at 37°C. Five to 7 days after splitting cells, cells were plated on L plates containing kanamycin and incubated at 37°C. Bigger colonies were isolated as suppressors of the slow growth phenotype of Δ*rodZ*. Chromosomal DNAs of wild-type and suppressor strains were extracted and sequenced by the Solexa system.

### Construction of strains carrying the *zipA*_*p56*_ suppressor mutation

To transfer the *zipA*_*p56*_ suppressor mutation, PCR fragments containing a cat resistance cassette flanked by an FLP recognition target site were inserted between the 1st and 2nd codons of the *yfeR* gene, which is downstream of *zipA*, in wild-type or the suppressor strain carrying the λ Red expression plasmid pKD46 (Datsenko and Wanner 2000). pKD3 was used as a template for PCR. Cm^R^ colonies were isolated after transformation of wild type or the suppressor, which has a mutation upstream of *zipA*, with PCR fragments to insert a cat resistance cassette in the *yfeR* gene. P1 phage were grown on a donor that carries mutations in the *yfeR* gene inserted with a cat resistance cassette with or without a mutation at the upstream of *zipA* gene and used to transduce BW25113 (WT), DS290 (Δ*rodZ*::*kan*), WM1074 (WT), or WM1125 (*ftsZ84*). Fresh transductants were restreaked on L plates containing Cm, and a Cm^R^ clone was selected, yielding DS645 (BW25113: Δ*yfeR*::*cat*), DS679 (BW25113: Δ*rodZ*::*kan*, Δ*yfeR*::*cat*), DS554 (BW25113: Δ*yfeR*::*cat*, *zipA*_*p56*_), DS631 (BW25113: Δ*rodZ*::*kan*, Δ*yfeR*::*cat zipA*_*p56*_), DS708 (WM1074: Δ*yfeR*::*cat*), DS709 (WM1074: Δ*yfeR*::*cat*, *zipA*_*p56*_), DS710 (WM1074: *ftsZ84*, Δ*yfeR*::*cat*), and DS711 (WM1074: *ftsZ84*, Δ*yfeR*::*cat zipA*_*p56*_). All of the mutation sites were sequenced and confirmed.

### Microscopy and measurements of the length and width

Cells were grown at 37°C in L broth or M9 medium containing 0.25% glucose overnight. The culture was diluted in L broth or M9 glucose and exponentially grown at 37°C. Cells were observed under an epifluorescence microscope (Zeiss, AXIO, Oberkochen, Germany). To take fluorescent images, delta vision microscopic system (Applied Precision, Issaquah, WA) was applied. The lengths of the long and short axes were measured as previously reported (Shiomi et al. 2008). Briefly, digital images were processed by the software Metamorph and changed into binary images to detect cell outlines. Each cell in the binary images was automatically measured for the length of the longest line (the long axis of the cell) and maximum breadth perpendicular to the longest line (the short axis of the cell).

### Isolation of saculli and vancomycin labeling

Cells were grown in M9 medium containing 0.25% glucose to log phase at 37°C and harvested by centrifugation for 10 min at room temperature. The pellet was resuspended in 3 mL of M9 medium containing 0.25% glucose and added dropwise to a tube containing 6 mL of 6% SDS. The samples were boiled for 3 h and incubated overnight at RT with gentle stirring. The next morning, the tubes were boiled for 1 h and sacculi were sedimented by ultracentrifugation at 173,970 g for 15 min at 30°C. The pellets were resuspended in 2.5 mL of 4% SDS in a closed tube and incubated in boiling water for 2 h. Sacculi were sedimented as above and washed with 1% SDS. Sacculi were resuspended with PBS containing 300 μg/mL α-chymotrypsin and incubated overnight at 37°C. Then, the next morning, the sacculi were pelleted as above, and resuspended in 1% SDS, incubated in boiling water for 2 h, and pelleted. The final pellet was resuspended in 100 μL H_2_O and stored at 4°C. The isolated sacculi were incubated with Van-FL (Molecular Probe, Carlsbad, CA) at room temperature for 1 h in the dark. The samples were washed with H_2_O and resuspended in H_2_O.

### Anti-FtsZ antiserum production

To raise antiserum against FtsZ, a 6x His (His_6_) tag was added to the *N*-terminus of FtsZ. KRX carrying pDS996 (a plasmid encoding *his*_*6*_*-ftsZ*) was grown in L broth supplemented with kanamycin and 0.25% glucose overnight. Cells were diluted 100 times into fresh L broth supplemented with kanamycin and incubated for 3 h at 37°C. Then cells were transferred to 25°C and incubated for 30 min. 0.1% rhamnose was added to the culture and the cells were incubated at 25°C overnight. Cells were harvested by centrifugation and resuspended in Buffer A (50 mmol/L NaPO_4_ [pH 7], 300 mmol/L NaCl, 8 mol/L urea) supplemented with one tablet of EDTA-free protease inhibitor cocktail (Roche, Penzberg, Upper Bavaria, Germany). The mixture was incubated for 1 h at 37°C, and centrifuged at 13,000 g for 20 min at 4°C. The supernatant containing His_6_-FtsZ was mixed with Talon metal affinity resin (Clontech, Mountain View, CA) equilibrated by Buffer A. The mixture was incubated at 4°C overnight. The mixture was centrifuged and the supernatant removed. The resin was resuspended in Buffer A and centrifuged. This step was repeated three times. His_6_-FtsZ was eluted by Buffer A containing 150 mmol/L imidazole. The protein was used to immunize rabbits, and anti-FtsZ antiserum was produced by Biogate (Gifu, Japan).

### Immunoblotting

Immunoblotting was performed with a blot using an antibody against ZipA (Geissler et al. 2003), which was provided by Dr. Margolin, or anti-FtsZ. Three clones for each strain were independently grown to mid log phase (OD_600_ = 0.6). The amounts of samples loaded onto electrophoresis gels were equally adjusted, monitoring by Coomassie Brilliant Blue (CBB) staining.

### Measurements of cell growth and cell number

Cells were grown at 37°C in L broth overnight. The culture was diluted 1:100 in fresh L broth and grown at 37°C. The Klett value was measured every 15 min. Culture samples (1 mL) were harvested at the indicated time points and stored on ice to measure cell number. Stored cultures (300 μL) were precipitated, washed with 500 μL of TE (pH 8), and resuspended with 90 μL of TE (pH 8). Next, 900 μL of 77% ethanol was added to the cells, followed by incubation at 4°C for 30 min. Following the incubation, 200 μL of 0.1 mol/L phosphate buffer (pH 9) was added to 20 μL of the samples. The samples were washed twice with 200 μL of 0.1 mol/L phosphate buffer (pH 9). The pellets were resuspended in 100 μL of 0.1 mol/L phosphate buffer (pH 9) and 100 μL of 3 μg/mL of FITC was added. The samples were incubated at 4°C overnight, then washed twice with 20 mmol/L PBS (pH 9), and resuspended in 1 mL of 20 mmol/L PBS. The number of cells in 250 μL of sample was measured by a cytometer (Cell Lab Quanta; BECKMAN, Indianapolis, IN).

### Real-time PCR

Total RNA was purified using an RNeasy Mini kit (QIAGEN, Venlo, Netherlands) following the supplied protocol. Then the RNA was reverse transcribed to obtain cDNA using a PrimeScript RT reagent kit (TAKARA, Shiga, Japan). Real-time PCR was performed and analyzed using SYBR Premix Ex Taq and a Thermal Cycler Dice Real Time System (TAKARA). mRNA was purified from three independent clones for each strain.

## Supporting Information

**Figure S1.** Morphology of the mutants grown in minimal medium.

**Figure S2.** Suppression of the cold-sensitive growth of the *rodZ* mutant by the *zipA_p56_* mutation.

**Figure S3.** Suppression of the swarming defect of the *rodZ* mutant by the *zipA_p56_* mutation.

**Figure S4.** Quantitative real-time PCR analysis of cells carrying WT or the mutant promoter of the *zipA* gene.

**Figure S5.** Growth and cell morphology of BW25113 (WT) or DS290 (Δ*rodZ*) cells carrying pBAD24 (vector; blue) or pDS1019 (pBAD24-*zipA*; red).

**Figure S6.** Viability of the *mreB* or *mrdA* mutants carrying the *zipA_p56_* mutation.

**Table S1.** Mass doubling time (min) of strains carrying suppressor mutations grown in L broth at 37°C.

**Table S2.** Strains and plasmids used in this study.
