# Supplementary material for: A mutation in the promoter region of zipA, a component of the divisome, suppresses the shape defect of RodZ-deficient cells
Source: Microbiologyopen. 2013 Aug 6;2(5):798–810. doi: 10.1002/mbo3.116 (PMC3831641; doi:10.1002/mbo3.116)
Supplement: Supplementary file 1 [file mbo30002-0798-SD1.doc]

**Figure legends to supplementary Figures**

**Figure S1. Morphology of the mutants grown in minimal medium.** Phase contrast images of DS645 (WT), DS554 (*zipA_p56_*), DS679 (*∆rodZ*), and DS631 (*∆rodZ zipA_p56_*) which were grown in M9 minimal medium containing 0.25% glucose at 37°C. Scale bar is 2 µm.

**Figure S2.** **Suppression of the cold-sensitive growth of the *rodZ* mutant by the *zipA_p56_* mutation.** (A) An overnight culture of DS645 (WT), DS554 (*zipA_p56_*), DS679 (*∆rodZ*), and DS631 (*∆rodZ zipA_p56_*) cells grown in L medium at 37˚C was diluted serially (from 10^-1^ to 10^-5^) and spotted onto L plates. The plates were incubated for 24 h at 25˚ for 48h or 37°C for 24h. (B) Phase contrast images of DS645 (WT), DS554 (*zipA_p56_*), and DS631 (*∆rodZ zipA_p56_*) cells grown in L medium at 25˚C to mid-log phase. Scale bar is 2 µm.

**Figure S3.** **Suppression of the swarming defect of the *rodZ* mutant by the *zipA_p56_* mutation.** An overnight culture of DS864 (WT), DS865 (*zipA_p56_*), DS866 (∆*cheA*), DS867 (∆*cheA* *zipA_p56_*), DS868 (∆*rodZ*), and DS870 (∆*rodZ* *zipA_p56_*) cells was spotted onto tryptone swarming plates (1% bacto tryptone, 0.5% NaCl, 0.3% agar). The plates were incubated at 33°C for 12 h. CheA is essential for chemotaxis so that cells are not chemotactic on the plate.

**Figure S4.** **Quantitative real-time PCR analysis of cells carrying WT or the mutant promoter of the *zipA* gene.** Panel shows the accumulation of PCR product (SYBR green fluorescence) during 45 cycles PCR amplifying *zipA* (triangles) and *gapA* (an internal control) (circles). Strains are DS645 (WT; blue), DS554 (*zipA_p56_*; red), DS679 (∆*rodZ*; orange), and DS631 (∆*rodZ* *zipA_p56_*; purple).

**Figure S5. Growth and cell morphology of BW25113 (WT) or DS290 (∆*rodZ*) cells carrying pBAD24 (vector; blue) or pDS1019 (pBAD24-*zipA*; red).** (A) Absorbance (OD_600_) was recorded automatically by a Bio-photorecorder (TVS 062CA, Advantech) every min at 37°C. WT (left) or DS290 (right) cells carrying pBAD24 (blue) or pDS1019 (red) were grown in L medium in the absence (open circles) or the presence of 0.2% arabinose (closed circles) at 37˚C. (B) Phase contrast images of WT or *∆rodZ* cells carrying pBAD24 or pDS1019 grown in L medium in the absence or presence of 0.2% arabinose at 37˚C. Scale bar is 5 µm.

**Figure S6. Viability of the *mreB* or *mrdA* mutants carrying the *zipA_p56_* mutation.** (A) An overnight culture of DS645 (WT), DS679 (∆*rodZ*), DS592 (∆*mreB*), DS594 (∆*rodZ* ∆*mreB*), DS600 (∆*mrdA*), DS602 (∆*rodZ* ∆*mrdA*), DS554 (*zipA_p56_*), DS631 (∆*rodZ* *zipA_p56_*), DS951 (∆*mreB* *zipA_p56_*), DS952 (∆*rodZ* ∆*mreB* *zipA_p56_*), DS953 (∆*mrdA* *zipA_p56_*) and DS954 (∆*rodZ* ∆*mrdA* *zipA_p56_*) grown in M9 minimal medium containing 0.25% glucose was diluted serially (from 10^-1^ to 10^-5^) and spotted onto L or M9 containing glucose plates. The plates were incubated for 24 h at 37°C. (B) Morphology of the mutants grown in M9 minimal medium containing 0.25% glucose at 37˚C. Scale bar is 2 µm.
