# Supplementary material for: A mutation in the promoter region of zipA, a component of the divisome, suppresses the shape defect of RodZ-deficient cells
Source: Microbiologyopen. 2013 Aug 6;2(5):798–810. doi: 10.1002/mbo3.116 (PMC3831641; doi:10.1002/mbo3.116)
Supplement: Supplementary file 2 [file mbo30002-0798-SD2.doc]

**Table. S1** Mass doubling time (min) of strains carrying suppressor mutations grown in L broth at 37 ˚C.

| Strain | Mutation(s) | ^a^Mass doubling time (min) |
| --- | --- | --- |
| BW25113 | Wild-type | 30 |
| DS290 | *∆rodZ*::*kan* | 47 |
| DS554 | *zipA_p56_* | 29 |
| DS631 | *∆rodZ*::*kan zipA_p56_* | 34 |

^a^Mass doubling time was calculated by measurement of OD600 of cells grown in L broth at 37˚C. Absorbance (OD_600_) was recorded automatically by a Bio-photorecorder (TVS 062CA, Advantech) every min at 37°C.

**Table S2.** Strains and plasmids used in this study.

| Strain | Description | Reference/ Source |
| --- | --- | --- |
| BW25113 | *rrnB, ∆lacZ478,* [*HsdR514*](http://www.shigen.nig.ac.jp/ecoli/pec/gatewayAction.do?chromosomalMarkerId=773)*, ∆(araBAD)567 ∆(rhaBAD)568, rph-1* | (Datsenko & Wanner, 2000) |
| JW2500 | Same as BW25113 but ∆*rodZ*::*kan* | (Ba*ba et a*l., 2006, Shio*mi et a*l., 2008) |
| DS290 | Same as BW25113 but ∆*rodZ*::*kan* | This study |
| DS343 | Same as BW25113 but ∆*rodZ* | This study |
| DS645 | Same as BW25113 but *∆yfeR*::*cat* | This study |
| DS679 | Same as BW25113 but *∆rodZ*::*kan, ∆yfeR*::*cat* | This study |
| DS554 | Same as BW25113 but *∆yfeR*::*cat, zipA_p56_* | This study |
| DS631 | Same as BW25113 but *∆rodZ*::*kan, ∆yfeR*::*cat, zipA_p56_* | This study |
| JD14320 | W3110 derivative, *∆mreB*::*Tn10kan* | (Mi*ki et a*l., 2008) |
| JD16067 | W3110 derivative, *∆mrdA*::*Tn10kan* | (Miki et al., 2008) |
| DS592 | Same as BW25113 but *∆mreB*::*Tn10kan* | This study |
| DS594 | Same as BW25113 but *∆rodZ,* *∆mreB*::*Tn10kan* | This study |
| DS600 | Same as BW25113 but *∆mrdA*::*Tn10kan* | This study |
| DS602 | Same as BW25113 but *∆rodZ, ∆mrdA*::*Tn10kan* | This study |
| DS951 | Same as DS592 but *∆yfeR*::*cat, zipA_p56_* | This study |
| DS952 | Same as DS594 but *∆yfeR*::*cat, zipA_p56_* | This study |
| DS953 | Same as DS600 but *∆yfeR*::*cat, zipA_p56_* | This study |
| DS954 | Same as DS602 but *∆yfeR*::*cat, zipA_p56_* | This study |
| WM1074 | MG1655 *∆lacU169* | (Shiomi & Margolin, 2007a) |
| WM1125 | Same as WM1074 but *ftsZ84* | (Shiomi & Margolin, 2007a) |
| DS708 | Same as WM1074 but *∆yfeR*::*cat* | This study |
| DS709 | Same as WM1074 but *∆yfeR*::*cat, zipA_p56_* | This study |
| DS710 | Same as WM1074 but *ftsZ84,* *∆yfeR*::*cat* | This study |
| DS711 | Same as WM1074 but *ftsZ84, ∆yfeR*::*cat zipA_p56_* | This study |
| Plasmid | Description | Reference/ Source |
| pDSW209 | P_trc_-*gfp*, pBR322 derivative, Amp^R^ | (Wei*ss et a*l., 1999) |
| pDSW210 | P_trc_-*gfp*, pBR322 derivative, Amp^R^ | (Weiss et al., 1999) |
| pWM2784 | Same as pDSW210 but carry a FLAG epitope sequence between *Eco*RI and *Sac*I sites | (Shiomi & Margolin, 2007b) |
| pDS156 | pDSW210-*ftsZ-gfp*, Amp^R^ | This study |
| pDS205 | pDSW209-*gfp-minD/minE*, Amp^R^ | This study |
| pWM2787 | pWM2784-*ftsA**, Amp^R^ | (Shiomi & Margolin, 2007a) |
| pBAD24 | P_BAD_, Amp^R^ | (Guzm*an et a*l., 1995) |
| pDS1019 | pBAD24-*zipA*, Amp^R^ | This study |
| pDS1391 | pBAD24-*cysZ*, Amp^R^ | This study |
| pDS996 | pET28a-*his6-ftsZ* | This study |

**References**

Baba, T., T. Ara, M. Hasegawa, Y. Takai, Y. Okumura, M. Baba, K. A. Datsenko, M. Tomita, B. L. Wanner & H. Mori, (2006) Construction of Escherichia coli K-12 in-frame, single-gene knockout mutants: the Keio collection. *Mol Syst Biol* **2**: 2006 0008.

Datsenko, K. A. & B. L. Wanner, (2000) One-step inactivation of chromosomal genes in Escherichia coli K-12 using PCR products. *Proc Natl Acad Sci U S A* **97**: 6640-6645.

Guzman, L. M., D. Belin, M. J. Carson & J. Beckwith, (1995) Tight regulation, modulation, and high-level expression by vectors containing the arabinose PBAD promoter. *J Bacteriol* **177**: 4121-4130.

Miki, T., Y. Yamamoto & H. Matsuda, (2008) A novel, simple, high-throughput method for isolation of genome-wide transposon insertion mutants of Escherichia coli K-12. *Methods Mol Biol* **416**: 195-204.

Shiomi, D. & W. Margolin, (2007a) Dimerization or oligomerization of the actin-like FtsA protein enhances the integrity of the cytokinetic Z ring. *Mol Microbiol* **66**: 1396-1415.

Shiomi, D. & W. Margolin, (2007b) The C-terminal domain of MinC inhibits assembly of the Z ring in Escherichia coli. *J Bacteriol* **189**: 236-243.

Shiomi, D., M. Sakai & H. Niki, (2008) Determination of bacterial rod shape by a novel cytoskeletal membrane protein. *Embo J* **27**: 3081-3091.

Weiss, D. S., J. C. Chen, J. M. Ghigo, D. Boyd & J. Beckwith, (1999) Localization of FtsI (PBP3) to the septal ring requires its membrane anchor, the Z ring, FtsA, FtsQ, and FtsL. *J Bacteriol* **181**: 508-520.
