# Supplementary material for: A mutation in the promoter region of zipA, a component of the divisome, suppresses the shape defect of RodZ-deficient cells
Source: Microbiologyopen. 2013 Aug 6;2(5):798–810. doi: 10.1002/mbo3.116 (PMC3831641; doi:10.1002/mbo3.116)
Supplement: Supplementary file 3 [file mbo30002-0798-SD3.pdf]

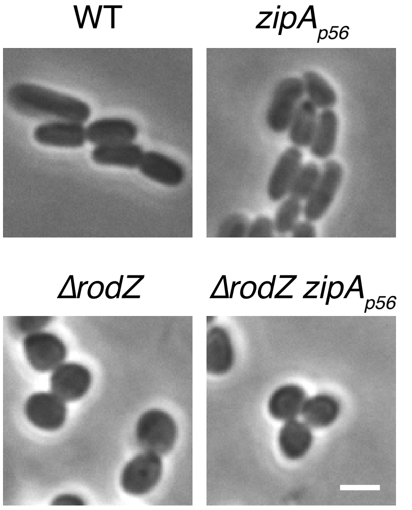

Figure S1, Shiomi and Niki

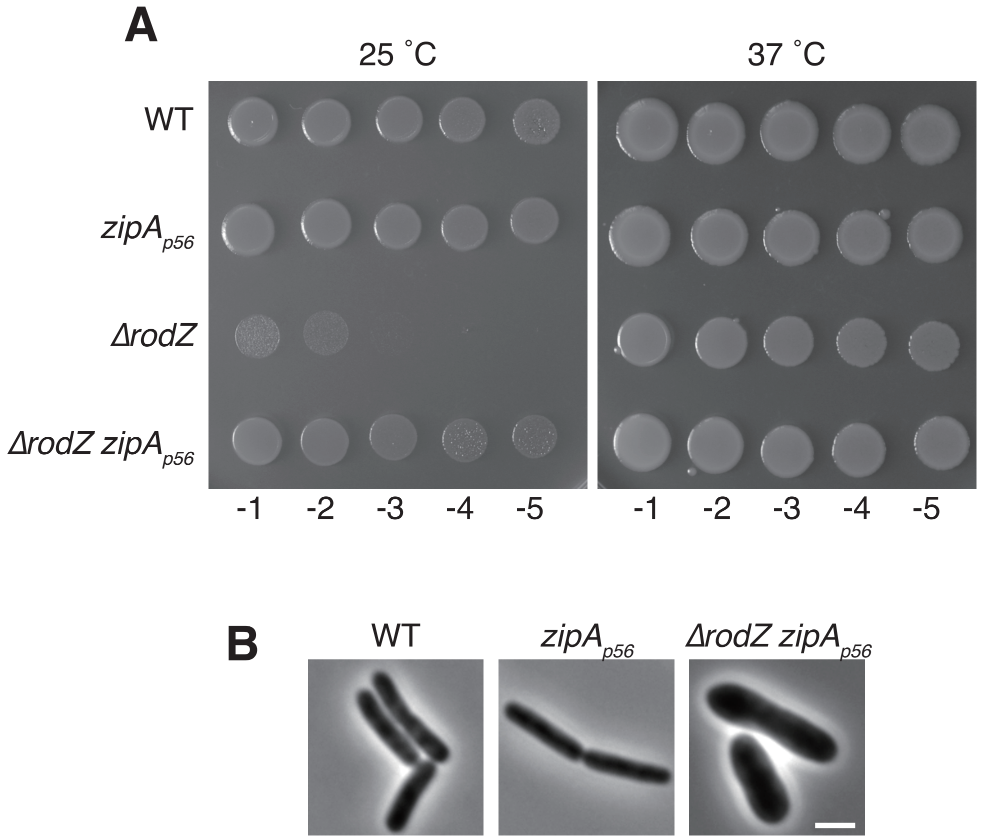

Figure S2, Shiomi and Niki

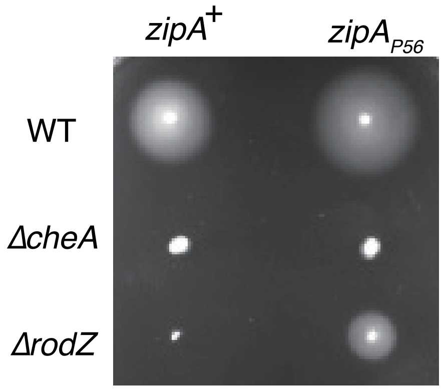

Figure S3, Shiomi and Niki

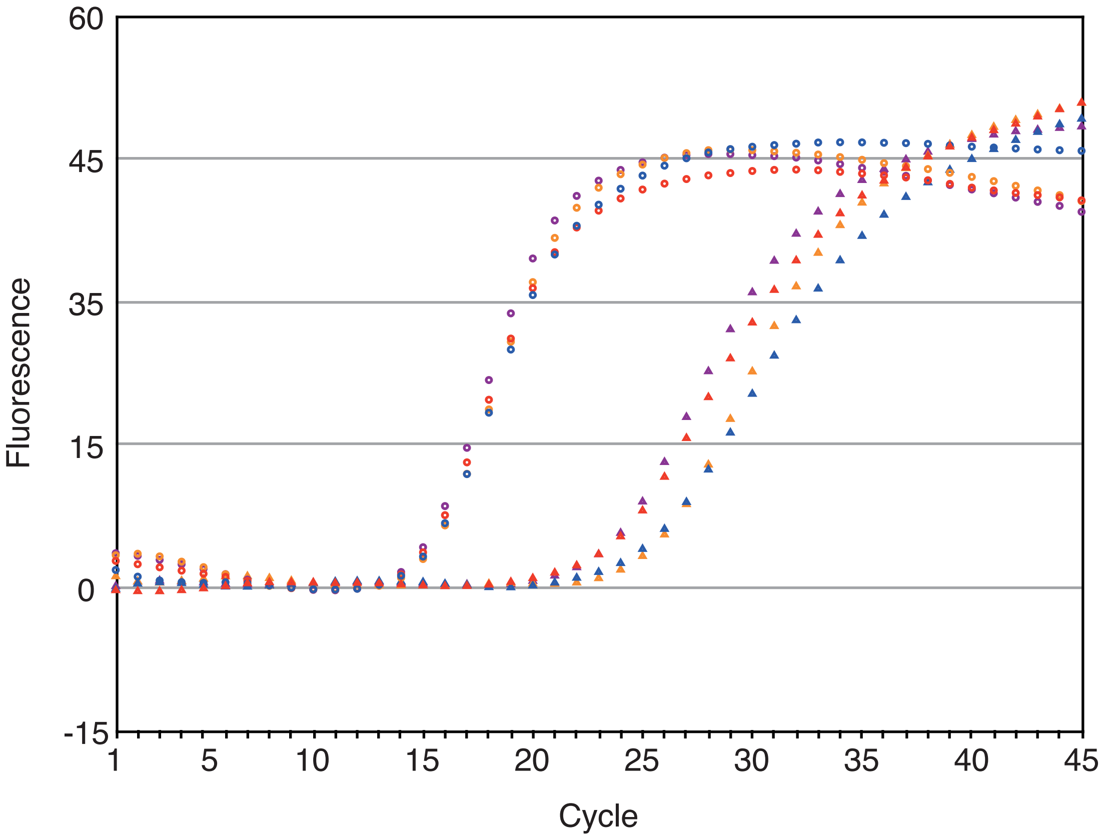

Figure S4, Shiomi and Niki

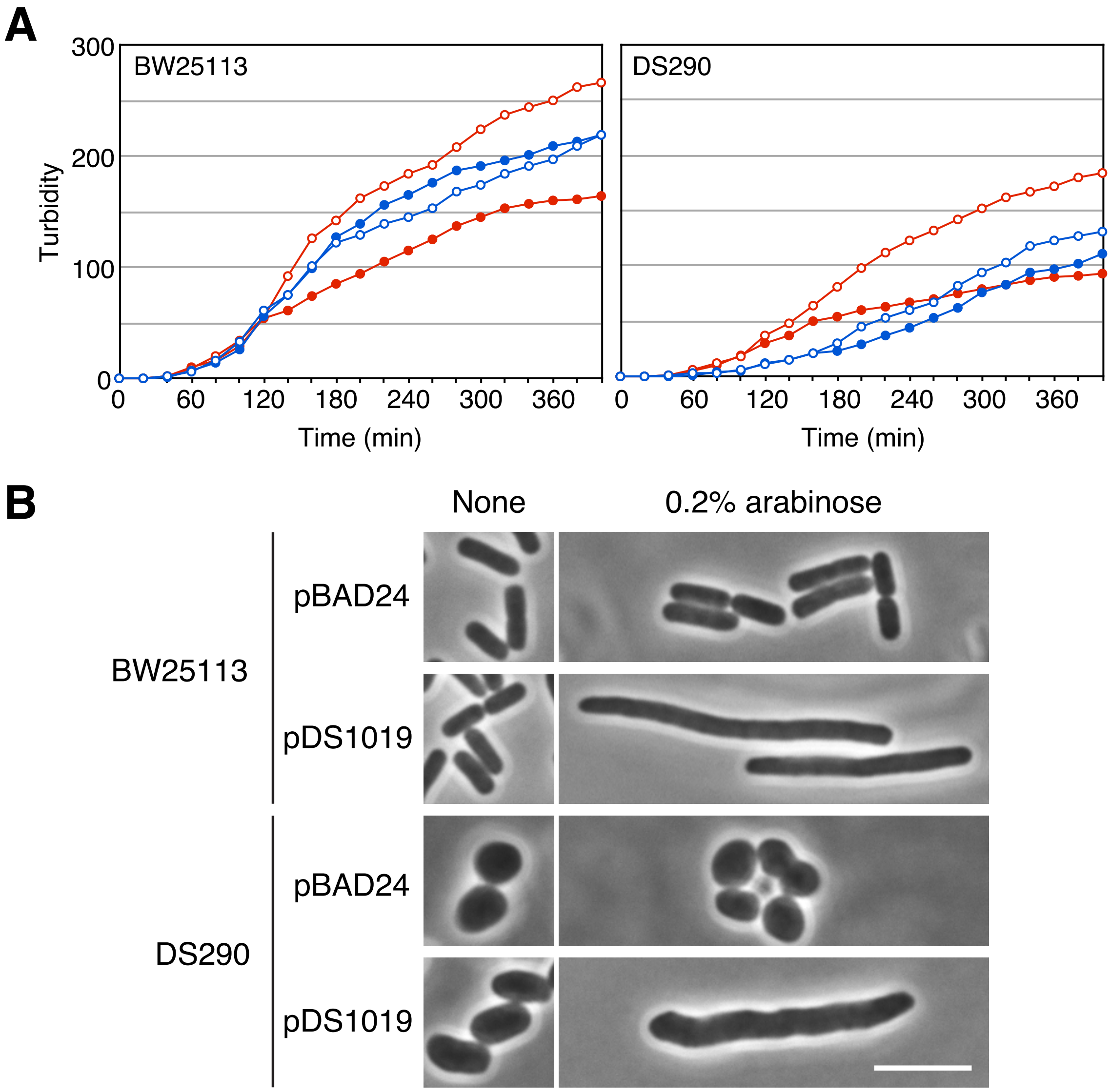

Figure S5, Shiomi and Niki

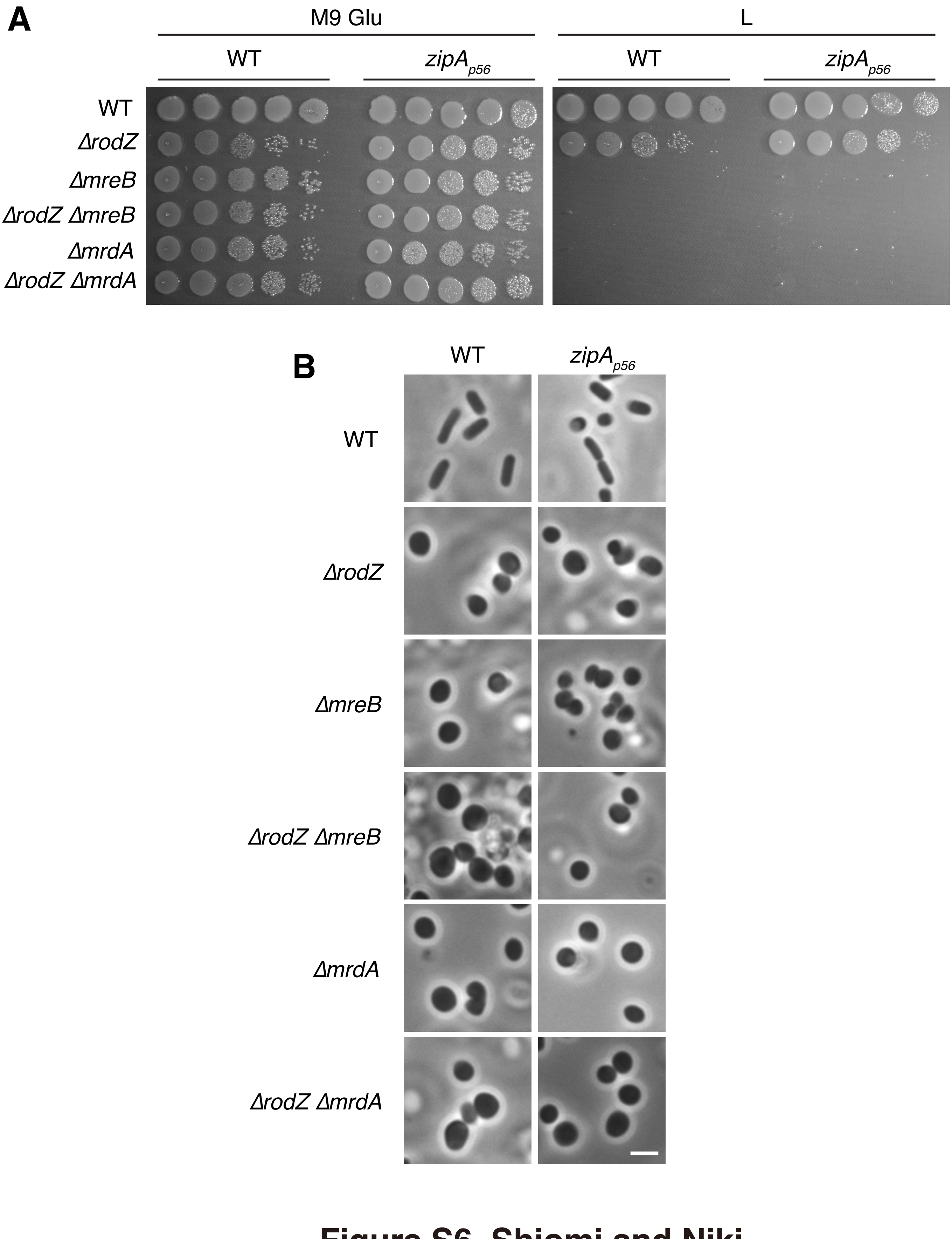

Figure S6, Shiomi and Niki
